# Complete genome sequencing of *Limosilactobacillus fermentum* NS2301G2 isolated from kimchi

**DOI:** 10.1128/mra.00665-25

**Published:** 2025-10-08

**Authors:** Ziyao Meng, Kiyeop Kim, Byung Wook Son, Jong Hoon Kim, Sejong Oh

**Affiliations:** 1 Division of Animal Science, Chonnam National University, Gwangju, Republic of Korea; 2 Research & Development Center, Nong Shim Co. Ltd., Seoul, Republic of Korea; Fluxus Inc., Sunnyvale, California, USA

**Keywords:** *Limosilactobacillus fermentum*, NS2301G2, complete genome sequence, tRNAs, rRNAs

## Abstract

*Limosilactobacillus fermentum* NS2301G2, isolated from fermented kimchi, underwent nanopore and Illumina sequencing, revealing a 2,077,637 base pair circular chromosome with 51.51% GC content, providing valuable genetic insights into lactic acid bacteria that contribute to food microbiology and probiotic research.

## ANNOUNCEMENT

**Fig 1:**
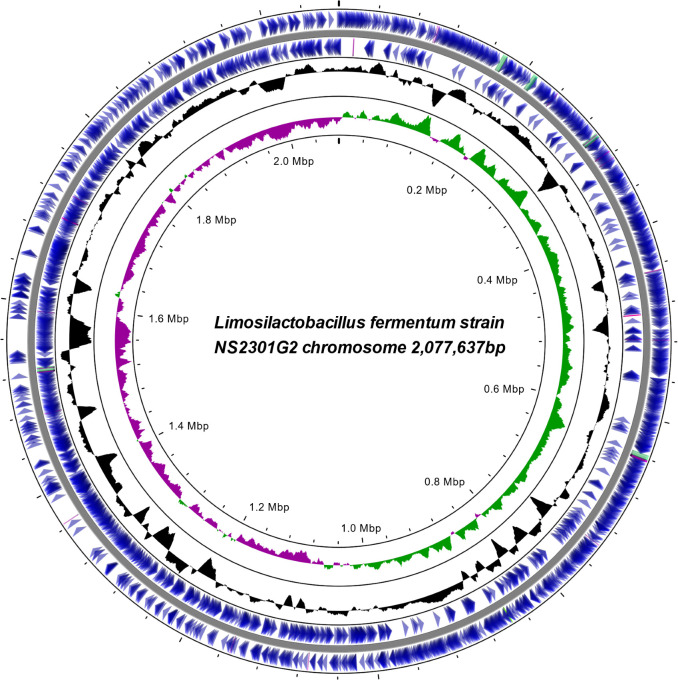
Circular chromosome map of *Limosilactobacillus fermentum* NS2301G2. Marked features are shown from the periphery to the center: protein coding sequences on the forward strand, protein coding sequences on the reverse strand, tRNA, rRNA, GC ratio, and GC skew. bp, base pair; G, guanine; C, cytosine; tRNA, transfer RNA; rRNA, ribosomal RNA.

Lactic acid bacteria (LAB) play essential roles in food fermentation and health. *Limosilactobacillus fermentum*, reclassified from the *Lactobacillus* genus (1), exhibits probiotic features such as acid and bile tolerance, cholesterol-lowering, and antioxidant activity (2). However, strain-specific genomic insights are limited, requiring further analysis. This study reports the complete genome sequencing of *L. fermentum* NS2301G2, isolated from fermented kimchi in Korea, using hybrid sequencing technologies that combined Illumina and Oxford Nanopore platforms. In a sterile environment, 10 g of kimchi was homogenized with 90 mL of 0.85% NaCl. Serial dilutions were plated onto de Man, Rogosa, and Sharpe (MRS) agar and incubated anaerobically at 37°C for 48–72 h. The isolate was cultivated in MRS broth at 37°C for 24 h under static conditions. Genomic DNA was extracted from 10 mL of culture using the DNeasy PowerSoil Pro Kit (QIAGEN, Hilden, Germany), following the manufacturer’s protocol. The quality and quantity of extracted DNA were assessed using a NanoDrop One spectrophotometer (Thermo Fisher Scientific, USA) and a Qubit 4 Fluorometer with the dsDNA BR Assay Kit (Thermo Fisher Scientific, USA), and integrity was confirmed by 1% agarose gel electrophoresis. Two genomic libraries were prepared for Illumina and Nanopore sequencing. For Illumina sequencing, 300 ng of genomic DNA was sheared to an average fragment size of approximately 400–450 bp using a Covaris M220 Focused-ultrasonicator (Covaris, Woburn, MA, USA) with microTUBE-50 AFA Fiber Screw-Cap tubes under the following parameters: peak incident power 75 W, duty factor 10%, 200 cycles per burst, and 64 s treatment time. Fragment size distribution was evaluated using a TapeStation 4200 system (Agilent Technologies, USA), confirming an average fragment size of ~400–450 bp. Libraries were then prepared using the TruSeq DNA Nano Prep Kit (Illumina, San Diego, CA, USA). Adapter trimming and quality filtering of Illumina reads were performed using Trimmomatic v0.39 (LEADING:10 TRAILING:10 SLIDINGWINDOW:4:20 MINLEN:200) (3). Reads were aligned to the phiX genome using Bowtie2 v2.3.5.1 (4) and filtered with SAMtools v1.9 (5). Sequencing was performed on the Illumina NextSeq2000 (Illumina) in the 300 bp paired-end mode, generating 5,255,888 reads (~1.55 Gb). After trimming, 2,296,328 high-quality pairs (87.38%) remained for downstream analysis. Nanopore libraries were prepared using the Ligation Sequencing Kit (Oxford Nanopore Technologies, Oxford, UK) and sequenced for 24 h on an R10.4.1 GridION flow cell, operated with MinKNOW v5.0.0 and basecalled using Guppy v6.0.6 (Oxford Nanopore Technologies, UK). No mechanical shearing or size selection was performed. Nanopore sequencing generated 65,416 reads (654 Mb), with a read N50 of 12,099 bp and average quality score of 16.7. Guppy v6.0.6 was used for basecalling. Reads were filtered using NanoFilt v2.8.0 (6) (Phred score <7, length <1,000 bp). All bioinformatics tools used default parameters unless noted. Trimmomatic v0.39 trimmed Illumina reads (4). Reads were aligned to the phiX genome using Bowtie2 v2.3.5.1 (5) and filtered with SAMtools v1.9 (6). Unicycler v0.4.8 assembled the genome using filtered Illumina and Nanopore reads (7). Genome annotation was performed with NCBI Prokaryotic Genome Annotation Pipeline v6.9 (PGAP v6.9) (8). Genome completeness was assessed using BUSCO v5.3.2 with the bacteria_odb10 lineage data set (9). The resulting assembly was a single circular contig of 2,077,637 bp with a GC content of 51.51%. Circularity was confirmed by trimming overlaps using Unicycler, and the genome was rotated to begin at the *dnaA* gene. The circular map (Fig. 1) was generated with Proksee (https://proksee.ca/) based on the PGAP-annotated GenBank file. Genome features are summarized in Table 1. BUSCO analysis indicated 99.2% completeness, with 0.8% fragmented and no missing single-copy orthologs, supporting the reliability of the assembled genome.

**TABLE 1:** Genome features of *Limosilactobacillus fermentum* NS2301G2

Statistics	*L. fermentum* NS2301G2
Total genome length (bp)	2,077,637
GC content (%)	51.51
Illumina Depth (X)	391.252
Nanopore Depth (X)	189.986
Genome coverage (x)	~581 total
CDS	2,059
tRNA	58
rRNA	15

## Data Availability

The complete genome sequence of *Limosilactobacillus fermentum* NS2301G2 has been deposited in GenBank under accession number CP165726. The 16S rRNA gene sequence is available under accession number PV926692. Raw sequencing data are available in the NCBI Sequence Read Archive (SRA) under accession numbers SRR34829517 (Illumina) and SRR34829516 (Oxford Nanopore). The BioProject accession number is PRJNA1141725 and the BioSample accession number is SAMN42955740. All entries are publicly available and consistently labeled as *L. fermentum* NS2301G2.
